# Surveys of Arboviruses Vectors in Four Cities Stretching Along a Railway Transect of Burkina Faso: Risk Transmission and Insecticide Susceptibility Status of Potential Vectors

**DOI:** 10.3389/fvets.2019.00140

**Published:** 2019-05-28

**Authors:** Lissy Parfait Eric Ouattara, Ibrahim Sangaré, Moussa Namountougou, Aristide Hien, Ali Ouari, Dieudonné Diloma Soma, Daouda Kassié, Abdoulaye Diabaté, Olivier Gnankiné, Emmanuel Bonnet, Valéry Ridde, Maurice Adja Akré, Florence Fournet, Kounbobr Roch Dabiré

**Affiliations:** ^1^Unité de Recherche-Paludisme et maladies Tropicales Négligées, Institut de Recherche en Sciences de la Santé, Bobo-Dioulasso, Burkina Faso; ^2^Centre Muraz, Bobo-Dioulasso, Burkina Faso; ^3^Institut Supérieur des Sciences de la Santé, Université Nazi Boni, Bobo-Dioulasso, Burkina Faso; ^4^ASTRE, CIRAD, Montpellier, France; ^5^UFR-Sciences de la Vie et de la Terre, Université Joseph-Ki Zerbo-Ouaga 1, Ouagadougou, Burkina Faso; ^6^Résiliences, IRD, Bondy, France; ^7^Department of Social and Preventive Medicine, School of Public Health (ESPUM), University of Montreal, Montreal, QC, Canada; ^8^Département D'Entomologie Médicale, Institut Pierre Richet, Bouaké, Côte d'Ivoire; ^9^MIVEGEC, IRD, Montpellier, France

**Keywords:** *Aedes aegypti*, *Stegomyia* indices, insecticide resistance, railway transect, Burkina Faso

## Abstract

**Background:** A severe outbreak of dengue occurred in Burkina Faso in 2016, with the most cases reported in Ouagadougou, that highlights the necessity to implement vector surveillance system. This study aims to estimate the risk of arboviruses transmission and the insecticide susceptibility status of potential vectors in four sites in Burkina Faso.

**Methods:** From June to September 2016, house-to-house cross sectional entomological surveys were performed in four cities stretching along a southwest-to-northeast railway transect. The household surveys analyzed the presence of *Aedes* spp. larvae in containers holding water and the World Health Organization (WHO) larval abundance indices were estimated. WHO tube assays was used to evaluate the insecticide susceptibility within *Aedes* populations from these localities.

**Results:** A total of 31,378 mosquitoes' larvae were collected from 1,330 containers holding water. *Aedes* spp. was the most abundant (95.19%) followed by *Culex* spp. (4.75%). *Aedes aegypti* a key vector of arboviruses (ARBOV) in West Africa was the major *Aedes* species found (98.60%). The relative larval indices, house index, container and Breteau indexes were high, up to 70, 35, and 10, respectively. *Aedes aegypti* tended to breed mainly in discarded tires and terracotta jars. Except in Banfora the western city, *Ae. aegypti* populations were resistant to deltamethrin 0.05% in the other localities with low mortality rate under 20% in Ouagadougou whereas they were fully susceptible to malathion 5% whatever the site. Intermediate resistance was observed in the four sites with mortality rates varying between 78 and 94% with bendiocarb 0.1%.

**Conclusions:** This study provided basic information on entomological indices that can help to monitor the risks of ARBOV epidemics in the main cities along the railway in Burkina Faso. In these cities, all larval indices exceeded the risk level of ARBOV outbreak. *Aedes aegypti* the main species collected was resistant to deltamethrin 0.05% and bendiocarb 0.1% whereas they were fully susceptible to malathion 5%. The monitoring of insecticide resistance is also important to be integrated to the vector surveillance system in Burkina Faso.

## Introduction

Since 2000, several outbreaks of dengue fever have been reported in West Africa from Senegal to Nigeria (1–8). In Burkina Faso, the occurrence of dengue fever outbreaks is not new. Seasonal epidemics had been reported decades ago both in Ouagadougou and Bobo-Dioulasso. In these regions, Zika and yellow fever viruses circulation was also observed (9, 10). Circulation of dengue viruses have been documented in Ouagadougou from 2006 to 2016 (11–13) with the occurrence of at least three serotypes that caused severe disease in 2016 (14, 15).

Even *Aedes aegypti* is assumed to transmitting dengue viruses (DENV) to humans, it is rare to find dengue viruses isolated from field mosquitoes' collections (16, 17). However, this species remains the main vector which transmits the four dengue virus serotypes worldwide (DENV1-4) (18). In West Africa several *Aedes* species have been associated to dengue transmission including *Ae. taylori, Ae. furcifer, Ae. luteocephalus, Ae. Vittatus*, and *Ae. aegypti* (10, 19, 20). A recent survey revealed the presence of *Ae. aegypti* and other sylvatic *Aedes* such as *Ae. luteocephalus, Ae. africanus*, and *Ae. cumminsi* in peripheral wooded areas of Bobo-Dioulasso city (21). *Aedes albopictus* originating from Southern Asia is another vector, and identified as one of the most competent and invasive species transmitting dengue and chikungunya viruses (22). It reached South and Central America (22) and Central Africa (23). This vector was recently reported in countries neighboring Burkina Faso, Côte d'Ivoire (24, 25) and Mali (26).

The domestic water containers favorable to vector development were the most identified risk factors for the emergence of dengue, Zika and chikungunya (27, 28). The breeding sites of *Ae. aegypti* are commonly found inside human dwellings, but also outside in many water holding containers. These containers are generated by human socioeconomic activities, including discarded tires (27, 28).

The World Health Organization (WHO) has defined specific entomological indices that correspond to transmission risk thresholds for ARBOV epidemic alerts. These indices set also areas to be targeted by vector control programs (29–31). Among them, the house index (HI), and the Breteau index (BI) are the most widely used (32, 33).

The control of ARBOV vectors is based on the suppression of breeding sites and the fogging of insecticides targeting adult stages when outbreak occurs. But insecticide resistance in *Ae. aegypti* populations can limit the success of chemical control. Evidence of this resistance was reported in West Africa (34–37). Many insecticides belonging to pyrethroids, carbamates, and organophosphates are widely used in Burkina Faso for the control of malaria vectors and from which *Anopheles gambiae* s.l. displayed high resistance status (38, 39). The official and recent dengue outbreaks occurred in Burkina in 2016 and 2017 and mostly reported in Ouagadougou and Bobo-Dioulasso from September to November. To riposte, insecticides belonging to organophosphates family were used in outdoor fogging against dengue vectors each year in the same months without any primary study to evaluate the susceptibility status of such vector populations prior to the intervention. Thus, arises the urgent need to conduct susceptibility test targetting the potential vectors of ARBOV collected in the study sites.

In Burkina Faso, the dengue vector surveillance has not yet been implemented despite a significant morbidity and mortality (15, 40) and above all with the risk of invasion of *Ae. albopictus*. Hence, in order to initiate the first large scale entomological surveys aiming to provide basic entomological data, we assessed the degree of *Stegomyia* dispersal in four major cities in Burkina Faso located along a railway. We also recorded the insecticide susceptibility within wild populations of *Ae. aegypti* collected from these sites tested against the main three families of insecticides usually used in Burkina in vector control program.

## Materials and Methods

### Study Sites

**Figure 1:**
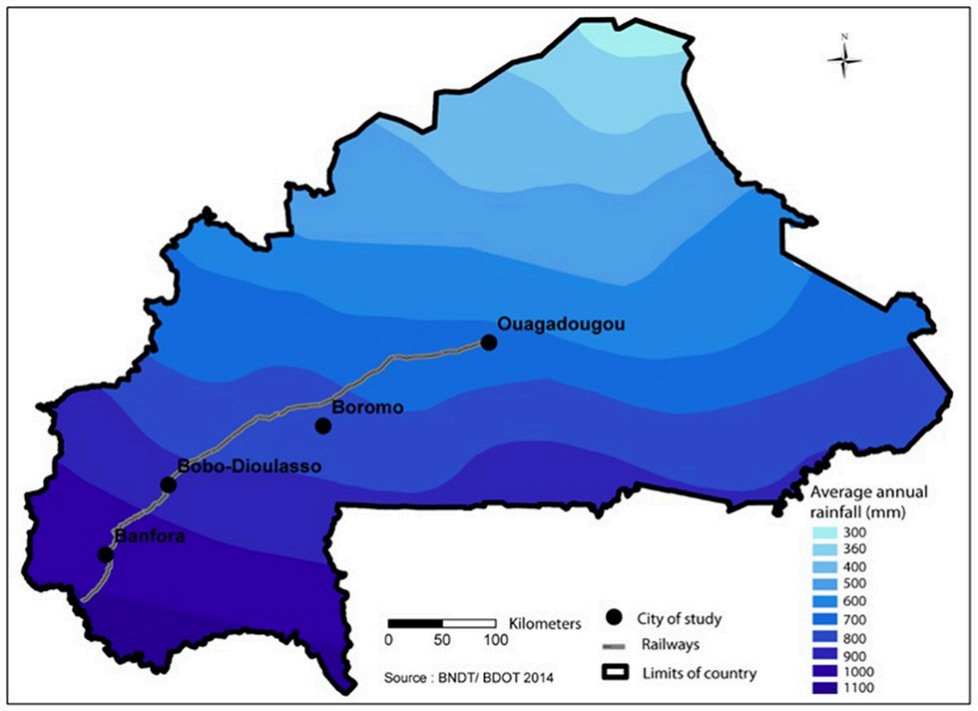
Map of Burkina Faso indicating the study towns following the railway.

The study was conducted following a transect across four railway towns Banfora, Bobo-Dioulasso, Boromo, and Ouagadougou (Figure 1). These towns were representative of the different environmental settings and human concentrations in the country. The traffic is particularly important in these commercial cities situated along the railway transect from Banfora to Ouagadougou. The study was performed between June and September 2016 during the rainy season, covering the most favorable period for *Culicidae* development. It was based on a house-to-house cross sectional entomological survey aiming to evaluate the *Stegomya* indices (29).

Ouagadougou (12°21′56″N, 1°32′01″W) is the capital city, located in the central part of the country. It had an estimated population of 2,293,635 people in 2016 (41). The climate is Sudano-Sahelian with average annual rainfall of 400–800 mm and average temperatures of around 36°C. Due to its aridity, large water dams have been built in the city. Vegetation is not dense except for a protected forest bordering the north side of the city. Two dengue outbreaks were reported in this city in 2016 and 2017 and annually dengue cases were reported from September to November form local people living the city.

Boromo (11°44′43.51″N, 2°55′48.22″W) is also central but south-west of Ouagadougou, with average annual rainfall reaching 900 mm and mean temperatures at 36°C. The city is more rural than urban with 37,066 inhabitants (41). It is a halfway between Bobo-Dioulasso and Ouagadougou (41).

Bobo-Dioulasso (11°10′59.999″N, 4°16′59.999″W) with about 780,846 inhabitants (41) is the second biggest city of the country, located in the West about 365 kilometers from Ouagadougou. The climate is more Sudanian, characterized by a relatively long rainy season from June to October with annual rainfall ranged from 1,000 to 1,200 mm and a dry season from November to May. The main rivers are the Kou and the Houet. Savannah-type vegetation dominates and the annual average temperature is about 34°C. Dengue cases were also reported each year since 2016 from local people living in this city.

Banfora (10°37′59.99″N, −4°46′0.01″ W) is a city, located in the extreme southwest of the country near the Côte d'Ivoire. The population size was estimated to be 148,581 inhabitants in 2016 (41). The eco-climatic characteristics are similar to those of Bobo-Dioulasso and the locality is crossed by an important permanent river, the Comoé.

### Study Design

In each city, two sampling areas were selected, except in Ouagadougou where only one area was selected because the second area was in an intervention site for dengue vector control. The first area was the central old quarter of each city which is usually more populated with concentrated housing system and the second one the peripheral quarter with extended landing occupation. Putting together the two sampling area were representative of the city. In each city 100 houses (cumulative of the two sampling areas) were randomly visited and the number of inhabitants per house was reported in a questionnaire sheet. In each locality, local people were trained for the prospecting of mosquito breeding sites and the collection of mosquito immatures. The geographical coordinates of all prospected houses were recorded using GPS.

### Mosquito Larvae Prospections

From each study area, in each sampled house, all containers found inside and outside human dwellings were inspected for the presence of water and occurrence of mosquito larvae. Each container was recorded by type, presence of water with or without larvae. In positive containers, mosquito larvae were collected with a pipette and put in plastic cups with the house identification number. The larvae collected were counted and classified by *Culicidae* genus. The mosquito breeding habitats were grouped into five types: plastic containers, metallic containers, terracotta jars, discarded tires, and other containers.

### Mosquito Species Composition

After field collections, plastic cups with mosquito larvae were transported to the insectary for rearing. All adults that emerged were identified to species under a microscope, using *Culicinae* identification keys (42, 43).

### WHO Insecticide Susceptibility Tests

As no formal diagnostic doses are established for *Aedes* susceptibility tests, we considered those for anopheline mosquitoes using the WHO tube protocol (44).

We used unfed female major mosquitoes of *Aedes aegypti* issued from field collection (F1) aged 2–5 days for WHO bioassay by determining their susceptibility to deltamethrin (0.05%), bendiocarb (0.1%), and malathion (5%) impregnated paper and compared to *Aedes aegypti* laboratory-reared, susceptible strain (Montpellier) considered as the lab susceptible reference strain (44). The mosquitoes were exposed to each insecticide for 60 min and then transferred to the observation tubes, fed with sugar pad and kept at 27–28°C to determine the mortality rate after 24 h. The three insecticides were selected as they represented each family of insecticides commonly used in public health in Burkina. Specifically, malathion had been used during the two dengue outbreaks in 2016 and 2017 and expected to be used in the future without any knowledge about any susceptibility status for *Ae. aegypti*.

### Data Analysis

WHO larval indices were used to assess the abundance of *Aedes* spp. based on house index (HI, percentage of houses positive for larvae), container index (CI, percentage of containers positive for larvae), and Breteau index (BI, number of positive containers per 100 houses). Container is referred to each individual recipient containing water that can constitute breeding habitat. Additional indices such as productivity index per person (PIP, the average number of L3 and L4 larvae per person related to the total persons leaving the household), productivity index per house (PIH, the average number of L3 and L4 larvae per house), and breeding preference ratio (BPR, the percentage of specific containers with L3 and L4 larvae divided by the total density of this specific containers) were also calculated. The risk of ARBOV transmission by *Ae. aegypti* is defined for dengue, chikungunya, yellow fever, and Zika viruses (31, 45, 46, 59).

The risk of ARBOV on each site was estimated using the WHO criteria as followed:

- An area where HI or BI or CI is <5, 20, and 3%, respectively, is considered unlikely to promote the transmission of dengue and chikungunya viruses by *Ae. aegypti*.
- An area where HI or BI or CI is <5, 4, and 3%, respectively, is considered unlikely to promote the transmission of Zika and yellow fever viruses by *Ae. aegypti*.
- An area where HI or BI or CI exceeds 5, 20, and 3%, respectively, is considered as presenting a high risk for *Ae. aegypti* to transmit dengue and chikungunya viruses.
- An area where the HI or BI or CI exceeds 5, 4, and 3%, respectively, is considered as presenting a high risk of *Ae. aegypti* transmitted Zika and yellow fever viruses.
- All analyses were performed using Statistical Package for Social Sciences (SPSS) version 20.0 at α = 0.05 level of significance. Chi-square tests were used to compare proportions relative to HI and CI between the cities.

## Results

### Numbers and Taxonomic Composition of Captured Immature Mosquitoes

In total 31,378 mosquito immatures were collected from 1,330 containers in the four cities. Among these, 95.19% were identified as *Aedes* spp., 4.75% as *Culex* spp., and the remaining (<1%) belonged to the *Anopheles* genus (Table 1).

**Table 1:** Number of *Culicidae* larvae collected by genus in the four towns.

***Culicidae***	**Banfora**		**Bobo-Dioulasso**		**Boromo**		**Ouagadougou**		**Total**	
	***n***	**%**	***n***	**%**	***n***	**%**	***n***	**%**	***n***	**%**
*Aedes*	8,685	99.54	7,169	97.66	10,895	95.80	3,121	79.23	29,870	95.19
*Culex*	37	0.42	172	2.34	463	4.07	817	20.74	1,489	4.74
*Anopheles*	3	0.03	0	0	15	0.13	1	0.03	19	0.06

*n, number; %, percentage*.

More *Aedes* larvae were found in suburban areas such as Banfora and Boromo than in urban areas of Bobo-Dioulasso and Ouagadougou.

The numbers of larvae of *Culex* mosquitoes varied greatly from one city to another, being more numerous in Ouagadougou and fewest in Banfora (817 vs. 37). Immature stages of *Anopheles* spp. were rare in the visited breeding sites.

Of the 3,139 *Aedes* mosquitoes that emerged at the adult stage in the insectary, only two species were identified: *Ae. aegypti* was the most frequent and abundant species at 98.60% (3,095/3,139) while *Ae. vittatus* compromised only 1.40% (44/3,139). *Ae. vittatus* was found in the cities of Bobo-Dioulasso and Boromo. No *Ae*. *vittatus* was reported in the collections from Banfora and Ouagadougou (Table 2).

**Table 2:** *Aedes* spp. adults emerged from larvae collected from breeding sites in the four towns.

**Towns**	***Ae. aegypti***			***Ae. vittatus***		
	**F**	**M**	***n* (%)**	**F**	**M**	**n (%)**
Banfora	440	345	785 (100)	0	0	0
Bobo-Dioulasso	387	384	771 (98.22)	10	4	14 (1.78)
Boromo	597	417	1,014 (97.13)	17	13	30 (2.87)
Ouagadougou	349	176	525 (100)	0	0	0
Total	1,773	1,322	3,095 (98.60)	27	17	44 (1.40)

*F, female; M, male; n, absolute numbers; (%), percentage*.

### Larval Indices and Breeding Site Types

The common larval indices (HI, CI, BI) varied between cities (Table 3). Relatively high HIs were recorded in all cities and were 79.2, 65, 65.8, and 24.6% in Banfora, Bobo-Dioulasso, Boromo, and Ouagadougou, respectively, and the HI values were significantly different among the different cities (χ^2^ = 113.5, *df* = 3, *P* < 0.0001).

**Table 3:** The WHO larvae abundance indices indicating the risk levels of arbovirus transmission in Burkina Faso.

**Towns**	**Total houses**	**Positives houses**	**Total containers**	**Positive containers**	**Total larvae**	**House index**	**Container index**	**Breteau index**
Banfora	120	95	356	167	8,685	79.17	46.91	139.17
Bobo-Dioulasso	120	78	313	87	7,169	65	27.80	72.50
Boromo	120	79	428	150	10,895	65.83	35.05	125
Ouagadougou	203	50	233	78	3,121	24.63	33.48	38.42

The CIs were estimated at 46.9, 27.8, 35.05, and 33.5% in Banfora, Bobo-Dioulasso, Boromo, and Ouagadougou, respectively, and there were significant differences among the survey cities (χ^2^ = 28.2, *df* = 3, *P* < 0.0001).

The BIs were estimated at 139.17, 72.50, 125, and, 38.42% in Banfora, Bobo-Dioulasso, Boromo, and Ouagadougou, respectively. It was relatively low in Ouagadougou, intermediate in Bobo-Dioulasso and high in the towns of Banfora and Boromo.

Out of a total of 1,330 breeding sites prospected, plastic containers, metallic containers and terracotta jars were estimated, respectively, at 28.27, 24.81, and 21.65% (Table 3). The most common breeding sites infested by *Aedes* mosquitoes were plastic containers (26.56%), discarded tires (22.82%), terracotta jars (22.61%), and metallic containers (20.95%). Significant differences existed among the positive containers (χ^2^ = 20.4, *df* = 4, *P* = 0.000426). Overall, the breeding preference ratio was higher with discarded tires (1.33), terracotta jars (1.04), and plastic containers (0.94).

The productivity index per person (PIPs) was estimated at 5.68, 3.54, 5.69, and 1.71 larvae (stage 3 and 4 larvae) per person, respectively, in Banfora, Bobo-Dioulasso, Boromo, and Ouagadougou (Table 4). The productivity index per house (PIH) was recorded at 72.38, 59.74, 90.79, and 15.37 larvae (L3 and L4) per house surveyed, respectively, in Banfora, Bobo-Dioulasso, Boromo, and Ouagadougou.

**Table 4:** Breeding Preference Ratio (BPR) of *Aedes* in the different types of containers.

**Container type**	**Number of containers with water**				**BPR (%Y/%X)**
	**Examined (X)**	**% X**	**With *Aedes* larvae (Y)**	**%Y**	
Plastic containers	376	28.27	128	26.56	0.94
Metallic containers	330	24.81	101	20.95	0.84
Terracotta jars	288	21.65	109	22.61	1.04
Discarded tires	229	17.22	110	22.82	1.33
Other containers	107	8.05	34	7.05	0.88
Total	1,330	100	482	100	

*Larvae, L3 and L4 stages*.

### Susceptibility Status of *Aedes aegypti* Populations

**Figure 2:**
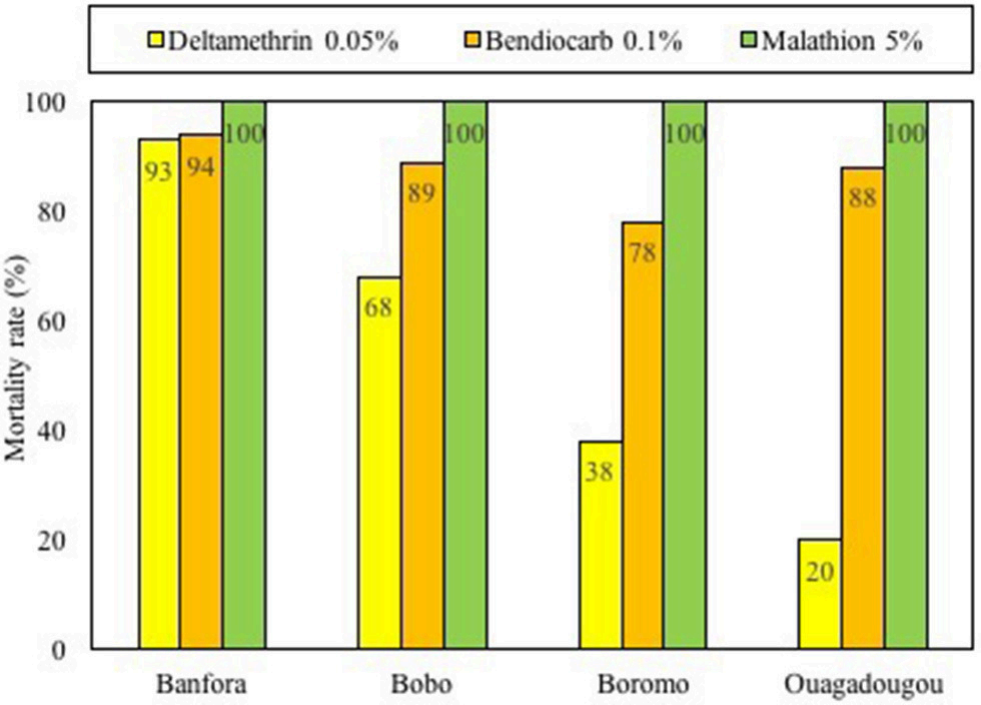
Insecticide susceptibility status of *Aedes aegypti* from Banfora, Bobo-Dioulasso, Boromo and Ouagadougou tested with deltamethrin 0.05%, bendiocarb 0.1%, and malathion 5%.

The WHO susceptibility tubes assays were performed with deltamethrin 0.05%, bendiocarb 0.1%, and malathion 5% with the populations of *Aedes aegypti*, the major vector collected in the four study sites (Figure 2). Most of the mosquito populations tested out of the four cities revealed high resistance level (<90% mortality) to deltamethrin 0.05%, with mortality rates ranging from 20 to 78%. Only *Ae. aegypti* populations from Banfora had showed moderate resistance to deltamethrin 0.05% with mortality rate reaching 94%. The tests with bendiocarb showed resistance in three populations: Ouagadougou, Boromo, and Bobo-Dioulasso with mortality rates ranging from 78 to 89%. Alike deltamethrin 0.05%, the mortality rate recorded with bendiocarb 0.1% in Banfora reached 94% indicating moderate resistance status.

*Aedes aegypti* populations were fully susceptible to malathion 5% reaching 100% in the four sites after 24 h exposure.

## Discussion

Our survey is one of the largest investigation of *Aedes* mosquitoes in urban areas in Burkina Faso. While several *Aedes* species have been associated to dengue and other ARBOV transmission in West Africa (10, 19, 20), *Ae. aegypti* appeared as the main potential ARBOV vector observed in our four study sites. Nevertheless, *Ae. vittatus* was also found in low proportion (<2%) in Bobo-Dioulasso and Boromo, especially in the periphery of these cities where rural settings were found, confirming that this species should be more sylvatic than *Ae. aegypti*.

An overview of dengue epidemiology in Africa during the past 5 years showed expansive hotpots in West Africa. Burkina Faso has experienced dengue epidemics reported by World Health Organization in urban areas of Ouagadougou in 2013, 2015, and 2016 (60). Moreover, in 2014, in Ouagadougou, the circulation of DENV-4 has been recorded for the first time in West Africa (14). In response to this epidemic, community awareness campaigns were carried out and insecticide spraying performed in peridomestic spaces of Ouagadougou. *Aedes aegypti* was assumed to be the primary vector, even though no DENV were isolated from mosquitoes collected during the epidemics. Our entomological surveys presented here confirmed *Ae. aegypti* is the main ARBOV vector species collected and so assumed to be the major vector of DENV in Burkina Faso and also likely to be the main potential vector of CHIKV, YFV, ZIKAV in cities of the country (47). As *Ae. albopictus* was recently reported in Côte d'Ivoire and Mali (25, 26), and because this species is very invasive, one must consider that it may reach Burkina Faso through the railway traffic. However, this species was not encountered among our collected specimens. One of the weakness of our study design was that it was not sufficiently elaborated to detect the occurrence of this species. We made a transect survey over a very short duration and so it may not have been sufficient to conclude this species absence. In Mali it took more than 4 years to formally confirm the presence of *Ae. albopictus* after its first detection.

Another limitation of our study is that the Breteau index, while commonly applied (45), is not considered very accurate a measure. In our case, its use is made difficult by the definition of the house. For example, a container may be shared by several households living in the same house. This situation was common in the survey areas, especially in densely populated districts. Consequently, the CI and indices based on the number of late instars (L3 and L4) mosquitoes per container or person are likely more accurate for assessing the risk of ARBOV transmission. Nevertheless, all these indices were estimated and compared to the thresholds considered by WHO. Considering the CI, more than 53% of 563 houses prospected were found to have containers with water in them. Additionally, all of the larval indices exceeded the critical level in all cities examined, thus they all had a high potential risk for DENV, CHIKV, YFV, and ZIKAV transmission (31, 45, 46, 59).

The risk of transmission of these arboviruses was highest in suburban areas of Boromo and Banfora, where we observed the highest larval and productivity indices. As the health system in Burkina is not efficient enough to assure accurate diagnosis of dengue disease in human patients, it is quite urgent to consider *Ae*. *aegypti* for vector control strategies. This surveillance program should also take into account semi-urban centers in order to prevent a spread of ARBOV epidemics.

*Aedes aegypti* is strongly associated with urban environments (48). In all the study sites we observed an accumulation of abandoned containers due to the absence of an effective waste management system. *Aedes aegypti* was found in all types of containers, but the discarded tires, terracotta jars and plastic containers were the most preferred breeding sites in our cities as previously observed in other contexts (49, 50). These containers preserve water, organic and mineral substances for a long time and allow vectors to reach adulthood (51). Thus, these containers could be considered as specific targets for the control of larvae through the elimination of larval sources by the community. The cities located along the railway transect are characterized by an abundance of human movement enabling the transport of vectors and/ or viruses from one site to another. All of these cities have also accumulated copious amounts of anthropic waste from their economic activities and limited waste management systems, including discarded tires, plastic and metallic and other domestic containers which are all suitable for the development of *Aedes*. Finally, land transport is expected to increase with the Abidjan-Niamey railway rehabilitation project, which will create an even more favorable environment for the circulation of vectors and arboviruses (52). If *Ae. albopictus* is assumed to be endemic in Mali and Côte d'Ivoire, it is crucial to initiate an entomological surveillance in these connecting cities. It is clear that the extension of ARBOV epidemics from these cities to other sites should be easier by their connectivity with other cities and countries.

This study was also conducted to evaluate the susceptibility of *Ae. aegypti* adults to deltamethrin 0.05%, bendiocarb 0.1%, and malathion 5%. Mosquitoes were exposed to the anopheline diagnostic doses recommended by WHO as no diagnostic doses are not yet established for *Aedes* mosquitoes. The objective was to facilitate the choice of insecticides to be used in spraying program by the ministry of health. The widespread use of insecticide in public health and agricultural practices had generally led to select insecticide resistance in several mosquitoes species (53). Multiple resistance to the main classes of insecticides was already observed in *Ae. aegypti* in the Caribbean Islands, Southeast Asia and South America (54–56). In sub-Saharan Africa some studies have reported resistance from *Ae. aegypti* to DDT 4% and pyrethroids with a decrease in susceptibility to carbamates and organophosphates compounds (34, 35, 37, 57).

*Aedes aegypti* resistance to deltamethrin 0.05% and bendiocarb 0.1% were observed in Ouagadougou, Boromo, and Bobo-Dioulasso. In Ouagadougou, these resistances could be worrying in the context of dengue epidemic reported in this city each rainy season since 2013. These epidemics and the particular case of 2016 have promoted not only the massive use of pyrethroid insecticides provided to control malaria vectors but also other new carbamate formulations that came on the market during this period. In all the surveyed cities from Banfora to Ouagadougou, resistance could be explained by the intensity of agricultural practices (cotton), market gardening and industrial activities, hence a massive use of pesticides. In addition, insecticide treated tools including impregnated bednets for malaria control, coils and repellents are widely used during the rainy season because of the strong culicidal nuisance. *Aedes aegypti* population collected in Banfora were moderately resistant to deltamethrin 0.05% and bendiocarb 0.1%. In this city, collections were made at the beginning of the rainy season when apparently the selection of resistant specimens should be low as the pressure of selection remained trivial before any large using of insecticides in agriculture occurs as this county is one of the most productive cotton area in Burkina. Our study also revealed that the wild populations of *Ae. aegypti* were found to be fully susceptible to malathion. Organophosphates are not frequently used in public health in Burkina Faso after the successes of onchocerciasis elimination campaigns. Even though these insecticides seem to not persist in long period in the environment (58), their intensive use for vector control could select and increase resistance in dengue vector populations (53). Thereby the need to monitor the evolution of resistance in these vector populations becomes relevant. Therefore, routine monitoring of insecticide resistance could be sustained in order to implement a management plan of insecticide resistance in *Ae. aegypti*.

## Conclusions

As long as a dengue vaccine and effective antiviral therapy will not be yet delivered, dengue prevention will be relied on the vector control. In order to promote such control in Burkina Faso, it is crucial to implement a vector surveillance system in at least some pilot areas for alert and intervention prospects. It is also important to initiate community education to inform people about the community engagement for ARBOV vector control and globally for integrated vector control importance.

Our results could be considered as preliminary on risk of transmission and insecticide susceptibility of *Ae. aegypti* population in a community, they call for the establishment of a national program for the control of arboviruses' diseases and their vectors in Burkina Faso.

## Ethics Statement

This study received approval from the local administrative and health authorities. Before performing the surveys, intended consent was obtained from households in each community.

## Author Contributions

KRD, MAA, and FF conceived and designed the experiments. LPEO, IS, AO, AH, and MN participated in the field collection and insecticide tests. DK drew the map. IS, LPEO, and KRD analyzed the data. KRD, AD, and FF contributed to material, analysis tools. IS, LPEO, and KRD wrote the paper. VR and EB corrected the manuscript. All authors read and approved the final version of the manuscript.

### Conflict of Interest Statement

The handling Editor declared a shared affiliation, though no other collaboration, with one of the authors FF. The remaining authors declare that the research was conducted in the absence of any commercial or financial relationships that could be construed as a potential conflict of interest.
